# Low-dose esketamine for the prevention of emergency agitation in children after tonsillectomy: A randomized controlled study

**DOI:** 10.3389/fphar.2022.991581

**Published:** 2022-12-20

**Authors:** Qi Li, Jiaming Fan, Wangping Zhang

**Affiliations:** ^1^ Department of Anesthesiology, The Second Affiliated Hospital of Jiaxing University, Jiaxing, China; ^2^ Department of Anesthesiology, Women and Children’s Hospital of Jiaxing University, Jiaxing, China

**Keywords:** esketamine, emergency agitation, children, tonsillectomy, general anesthesia

## Abstract

**Background:** Emergency agitation is a common postoperative complication in pediatric patients after general anesthesia. The aim of this study was to explore the effects of a low dose of esketamine on emergency agitation in children following tonsillectomy.

**Materials and Methods:** Eighty children were recruited prospectively to this study and divided into the esketamine group and the control group (40 cases in each group). The induction and maintenance of anesthesia were the same in both groups. At the end of surgery, the esketamine group received 0.25 μg/kg esketamine, while the control group received the same volume of normal saline. The extubation time, time to eye opening, Ramsay sedation scale and time to discharge from the post-anesthesia care unit (PACU) were recorded during post-anesthesia care unit. Postoperative complications, such as emergency agitation, respiratory depression, hypertension, tachycardia, nightmares, nausea, and vomiting, were also recorded.

**Results:** The incidence of emergency agitation was lower in the esketamine group compared with that in the control group (5% *vs*. 27.5%, *p* = 0.006). The time to eye opening was longer in the esketamine group than in the control group (17.2 ± 2.7 *vs*. 15.5 ± 2.3 min, *p* = 0.005). However, the extubation time and time to discharge from PACU were similar between the two groups.

**Conclusion:** Low-dose of esketamine decreases the incidence of emergency agitation in children after tonsillectomy without delaying extubation time and increasing the postoperative side effects. (www.chictr.org.cn, registration number: ChiCTR2100054178).

## 1 Introduction

Emergence agitation is a well-known phenomenon in children after general anesthesia, (Mohkamkar et al., 2014; Cao et al., 2016), especially in those undergoing adenotonsillectomy. (Fattahi-Saravi et al., 2021). The incidence of emergence agitation varies from 0.25% to 90.5%. (Lee and Sung, 2020). Emergence agitation might cause injury, accidental removal of intravenous cannulation, self-extubation, post-operative wound bleeding, and increase the nursing requirements in the post-anesthesia care unit (PACU).. (Zhu et al., 2021).

Intravenous administration of a subhypnotic dose of midazolam, propofol, and ketamine combined with fentanyl is usually performed to decrease the incidence of emergence agitation in children undergoing sevoflurane anesthesia. (Chen et al., 2010). Although intravenous administration of fentanyl at the end of surgery could reduce the incidence of emergence agitation in children undergoing general anesthesia, it was associated with a prolonged PACU stay and an increased incidence of postoperative nausea or vomiting. (Kim et al., 2017). The literature reported that dexmedetomidine decreased the incidence of emergence agitation in children after general anesthesia, but it was accompanied by delayed extubation time. (Yang et al., 2020).

Ketamine is an antagonist of the N-methyl-d-aspartate receptor. In recent years, it has been used widely in clinical anesthesia, pain management, and resistant depression. (Frey et al., 2019; Salloum et al., 2019). Esketamine is a more potent *S*-isomer of ketamine. Its potency is approximately two times higher than that of ketamine, but with fewer side effects. (Jonkman et al., 2018; Wang et al., 2019). Moreover, esketamine offers a shorter recovery time and orientation recovery time compared with ketamine. (Wang et al., 2019). To date, there have been no studies on the use of esketamine to prevent emergency agitation. Therefore, the present study aimed to investigate the efficacy of esketamine in reducing emergency agitation in children undergoing tonsillectomy.

## 2 Materials and methods

This study was conducted in accordance with the Declaration of Helsinki and was approved by the Ethical Committee of the Jiaxing Children’s Hospital (approval number: 201836, Chairman: Prof L. Xia). Written informed consent was obtained from the guardians of the children (www.chictr.org.cn, registration number: ChiCTR2100054178).

From 1 December 2021 to 1 March 2022, a total of 80 children undergoing tonsillectomy with American Society of Anesthesiologists (ASA) stage I–II, weighing between 10 and 50 kg, and aged from 2 to 7 years were included this study. Children with cardiovascular diseases, psychosis, or mental diseases were excluded. The children were randomly divided into the control group and the esketamine group (40 cases in each group). The investigators, anesthesiologists, surgeons, and nurses were blinded to the group allocation.

The children, who did not receive premedication, were fasted for 8 h. After entering the operating center, venous access was established. The electrocardiogram, pulse oxygen saturation (S_P_O_2_), non-invasive blood pressure, and heart rate (HR) were monitored. Anesthesia was induced using intravenous fentanyl at 3 μg/kg, propofol at 3 mg/kg, and cis-atracurium at 0.12 mg/kg. Endotracheal intubation was performed under direct laryngoscopy. Subsequently, the lungs were ventilated in the pressure-controlled ventilation mode. The ventilation parameters were set as follows: a driving pressure of 15–18 cmH_2_O, a breathing rate of 14–20 breaths/min, an oxygen flow rate of 2 L/min, a fraction of inspired oxygen of 0.5, an inspiratory expiratory ratio of 1:1.5, and a positive end-expiratory pressure of zero.

The driving pressure was adjusted to keep the end-tidal carbon dioxide partial pressure (P_ET_CO_2_) between 35 and 45 mmHg. Anesthesia was maintained using 2%–4% sevoflurane to keep the systolic blood pressure within a 20% range of baseline. Anesthetics were stopped 5 min before the end of surgery. The esketamine group received 0.25 mg/kg esketamine (Jiangsu Hengrui pharmaceutical company, Jiangsu, China) at the end of surgery and the control group received the same volume of normal saline, after which all the children were transferred to the PACU. These medications were prepared by nurses who were blinded to the grouping.

The systolic blood pressure (SBP), diastolic blood pressure (DBP), and HR were recorded at 3 min after drug administration. The extubation time, time to eye opening, Ramsay sedation scale (RSS) in the PACU, and time to discharge from the PACU were also noted. The adverse events (emergency agitation, respiratory depression, tachycardia, hypotension, nightmares, nausea, and vomiting) were recorded. The endotracheal tube was extubated when the tidal volumes were >6 ml/kg, S_P_O_2_ was >96%, and P_ET_CO_2_ was <45 mmHg during air inhalation. The standards of discharge from the PACU were as follows: ***a.*** Awake. ***b.*** Air breathing SpO_2_ > 94%. *c*. Moving the arms, legs, and head with commands. ***d.*** Breathing with a normal respiratory rate. Respiratory depression was defined as SpO_2_ < 94% whilst receiving oxygen and a breathing rate <10 times per minute. Hypertension was defined as an SBP above 20% from the baseline values and tachycardia was defined as an HR above 20% from the baseline values. The children were given 1 mg/kg propofol by intravenous injection if emergency agitation occurred.

The level of sedation was assessed using the Ramsay sedation scale (RSS) (1, patients were anxious, agitated, or restless, or both. 2, patients were cooperative, oriented, and tranquil. 3, patients responded to commands only. 4, patients were asleep but had a brisk response to a light glabellar tap or loud auditory stimulus. 5, patients were asleep with a sluggish response to a light glabellar tap or loud auditory stimulus. 6, patients were asleep and not responsive). Emergency agitation was defined as an RSS value of 1.

### 2.1 Statistical analysis

In this study, the primary outcome was the incidence of emergency agitation. *A priori* power analysis using two-sided analysis with an α error of 0.05 and a power of 0.8 showed that 32 patients were needed to detect a statistical difference in the incidence of emergency agitation between the two groups for this study. The sample size was increased to 40 to allow for dropout in each group. Data analysis was performed with the SPSS 20.0 statistical software (IBM Corp., Armonk, NY, United States). Data are presented as the mean ± standard deviation. Comparison of the numerical variables between the two groups was performed using a *t-*test for independent samples. The categorical data were compared using a Chi-squared test or Fisher’s exact test. *p-*values <0.05 were considered statistically significant.

## 3 Results

**FIGURE 1:**
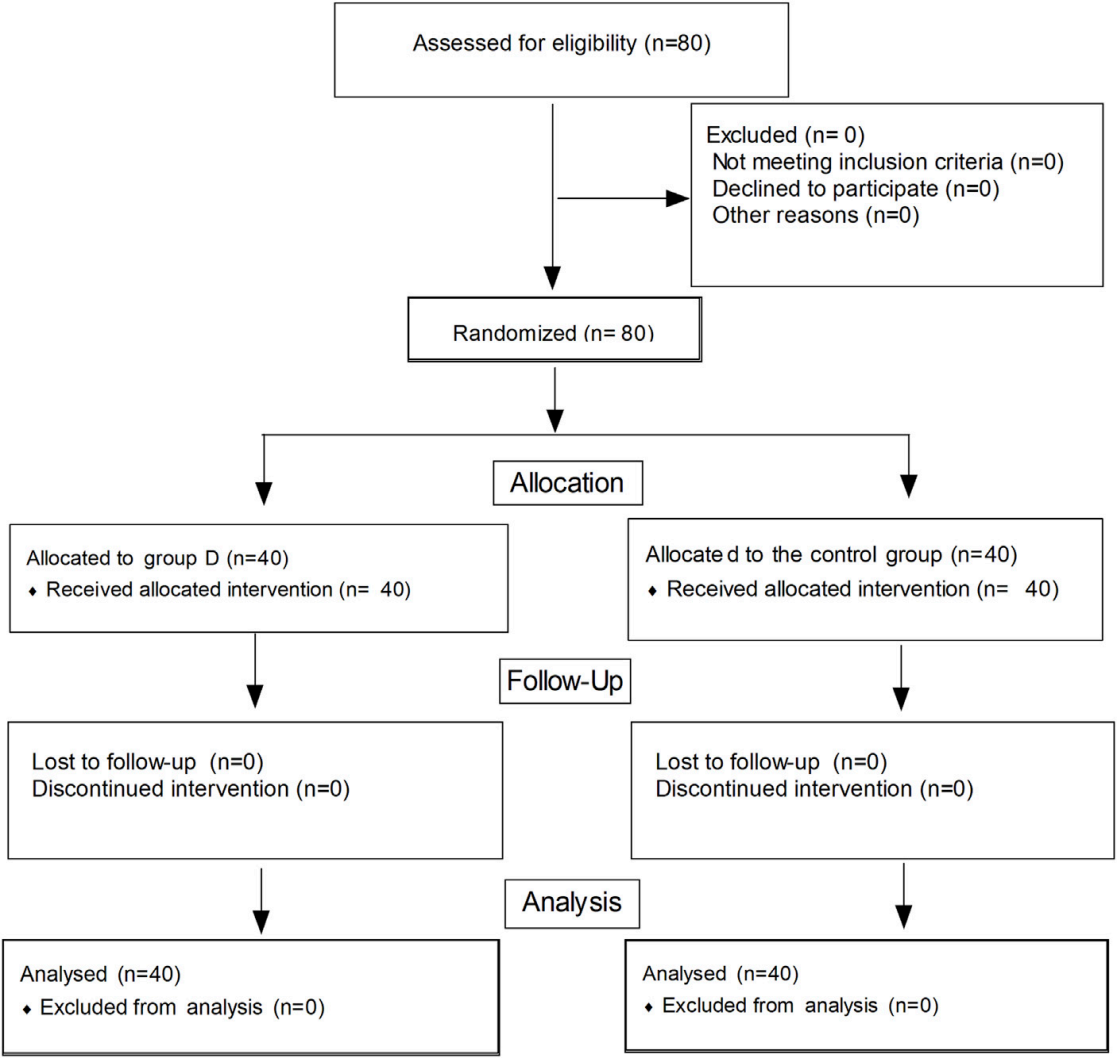
Flow diagram of the study.

**FIGURE 2:**
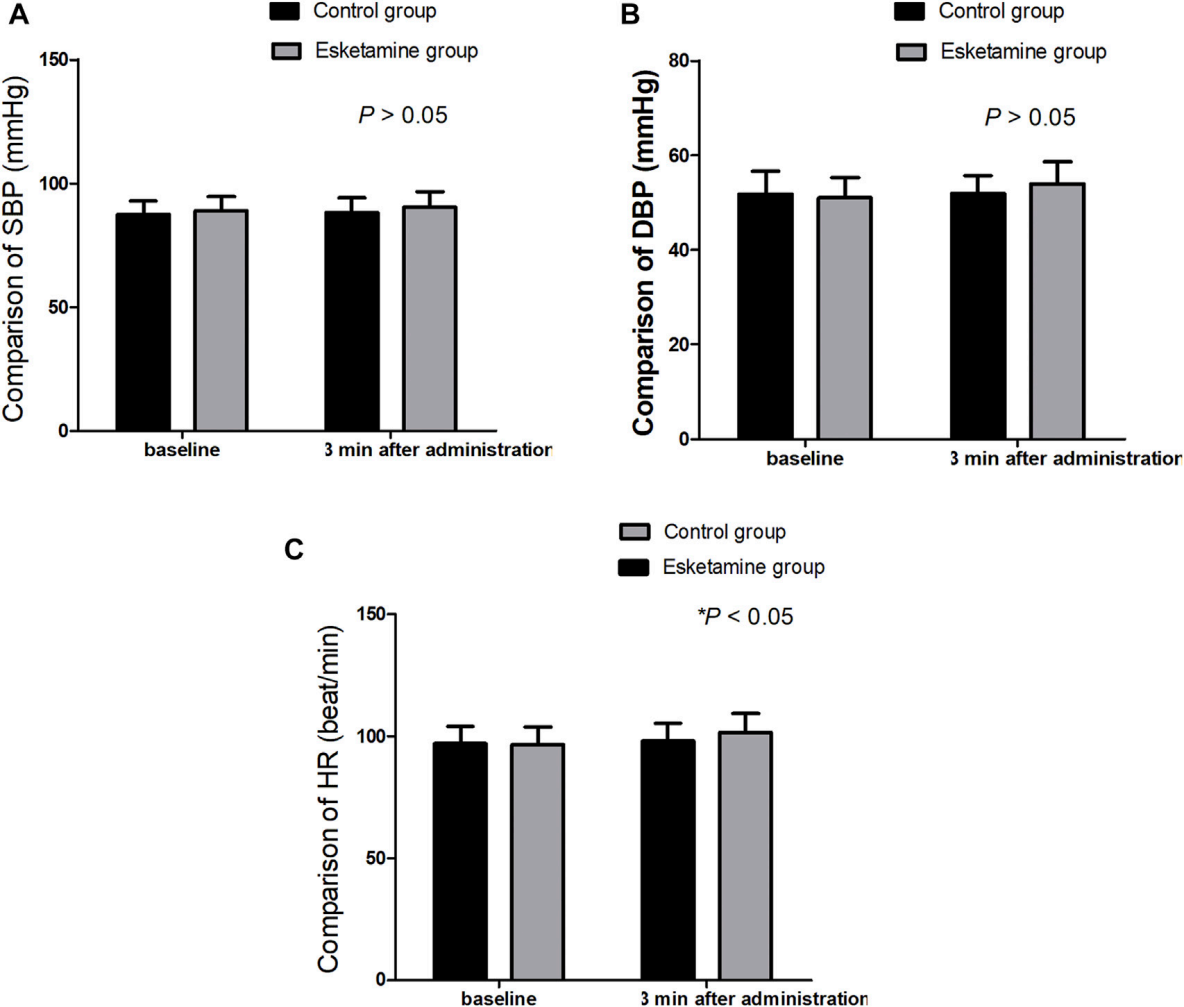
**(A–C)** Comparison of SBP, DBP, and HR between the two groups, ^*^ *p* < 0.05.

A total of 80 children were enrolled in this trial (Figure 1). There were no significant differences in terms of the age, sex, weight, body mass index, duration of operation, and duration of anesthesia between the two groups (*p* > 0.05) (Table 1). The extubation time and time to discharge from the PACU were similar between the two groups (*p* > 0.05); however, the time to eye opening was longer in the esketamine group than in the control group (17.2 ± 2.7 *vs*.15.5 ± 2.3 min, *p* = 0.005) (Table 1). The SBP and DBP at 3 min after drug administration were similar between the two groups (*p* > 0.05); however, the HR at 3 min after drug administration was greater in the esketamine group than in the control group (*p* = 0.042) (Figure 2).

**TABLE 1:** Data of children (*n* = 40).

Index	Esketaminegroup	Control group	*p*-Value
Age (year)	4.6 ± 1.0	4.5 ± 1.3	0.622
Gender (male/female)	22/18	21/19	0.823
Weight (kg)	17.2 ± 1.9	16.7 ± 2.1	0.305
Body mass index (kg/m2)	23.4 ± 3.5	23.6 ± 3.2	0.861
Duration of anesthesia (min)	54.9 ± 6.8	56.4 ± 6.3	0.328
Duration of surgery (min)	41.4 ± 7.7	42.4 ± 8.2	0.567
Extubation time (min)	11.5 ± 2.3	10.4 ± 2.4	0.129
Time to eye opening (min)	17.2 ± 2.7	15.5 ± 2.3	0.005
Time to discharge from PACU (min)	40.7 ± 7.8	38.6 ± 7.9	0.223

Data are expressed as the mean +standard deviation or number. PACU: post-anesthesia care unit.

There were two case of emergency agitation in the esketamine group, while 11 cases of emergency agitation were reported in the control group (5% *vs*. 27.5%, *p* = 0.006). There were no statistical differences in the incidence of respiratory depression, hypotension, tachycardia, nightmare, nausea, and vomiting between the two groups (*p* > 0.05) (Table 2).

**TABLE 2:** Comparison of side effects in children (*n* = 40).

Index	Esketamine group	Control group	*p*-Value
Emergency agitation n)	2 (5%)	11 (27.5%)	0.006
Respiratory depression n)	0	0	—
Hypertension n)	0	0	—
Tachycardia n)	1	0	0.999
Nightmare n)	1	0	0.999
Nausea and vomiting n)	0	1	0.999

Data are expressed as number (percent).

## 4 Discussion

This study showed that low-dose esketamine reduced the incidence of emergency agitation in children after tonsillectomy without delaying extubation time and increasing postoperative side effects.

Esketamine can produce an analgesic effect by activating the N-methyl-d-aspartate (NMDA) receptor. (Jonkman et al., 2018). In the present study, the incidence of emergency agitation in the PACU was lower in the esketamine group than in the control group. There are many factors related to emergency agitation in patients undergoing general anesthesia, including the use of inhalation anesthetics, rapid recovery, type of surgical procedure, postoperative pain, hypoxia, and airway obstruction. (Kim et al., 2015; Wei et al., 2021). Postoperative pain or discomfort is the main factor in emergency agitation. (Zhong et al., 2018). Esketamine could provide long-term sedation and analgesia, and good sedation and analgesic effects could decrease the incidence of emergency agitation in children after tonsillectomy. Chen, et al. (Chen et al., 2010) reported that low doses of midazolam-fentanyl or propofol-fentanyl were both effective to decrease the incidence of emergency agitation in children undergoing cataract extraction after general anesthesia, without significantly delaying recovery time and time to PACU discharge. Lee, et al. (Lee et al., 2010) found that ketamine was decreased the incidence of emergency agitation in children undergoing tonsillectomy and adenoidectomy after sevoflurane general anesthesia without increasing the extubation time and delivery time from the PACU. Their findings agreed with those of the present study. Ni, et al. (Ni et al., 2015) reported that intravenous dexmedetomidine significantly reduced the incidence of emergency agitation; however, it increased the time to eye-opening and the time to discharge from the PACU.

In the present study, the extubation time and time to discharge from the PACU were similar between the two groups; however, the time to eye opening was longer in the esketamine group compared with that in the control group. A study reported that esketamine countered opioid-induced respiratory depression, (Jonkman et al., 2018), and the clinical dose of esketamine did not cause respiratory depression; therefore, it did not result in prolongation of the extubation time. Low-dose esketamine did not result in extension of the time to discharge from the PACU. This effect was dose-dependent and a high dose of esketamine led to delayed time to PACU discharge. In this study, we found that esketamine increased the time to eye opening, which was associated with the anesthetic effect of esketamine.

No significant differences were observed in the incidence of respiratory depression, hypertension, tachycardia, nightmares, nausea, and vomiting between the two groups in the present study. Hypertension, tachycardia, and nightmares are common side effects of esketamine administration. Low-dose esketamine did not increase the incidence of these side effects. In our study, low-dose esketamine (0.25 mg/kg) did not affect the blood pressure, but led to HR increases (*p* < 0.05).

### 4.1 Limitations

Nightmares cannot be assessed accurately in children. Furthermore, a large sample study is needed to investigate the side effects of esketamine.

## 5 Conclusion

This study indicated that low-dose esketamine reduced the incidence of emergency agitation in children undergoing tonsillectomy without delaying extubation time and increasing the postoperative side effects.

## Data Availability

The original contributions presented in the study are included in the article/supplementary materials, further inquiries can be directed to the corresponding authors.
